# Supplementary material for: Dietary Docosahexaenoic Acid (DHA) and Eicosapentaenoic Acid (EPA) Operate by Different Mechanisms to Modulate Hepatic Steatosis and Hyperinsulemia in fa/fa Zucker Rats
Source: Nutrients. 2019 Apr 24;11(4):917. doi: 10.3390/nu11040917 (PMC6521162; doi:10.3390/nu11040917)
Supplement: Supplementary file 1 [file nutrients-11-00917-s001.pdf]

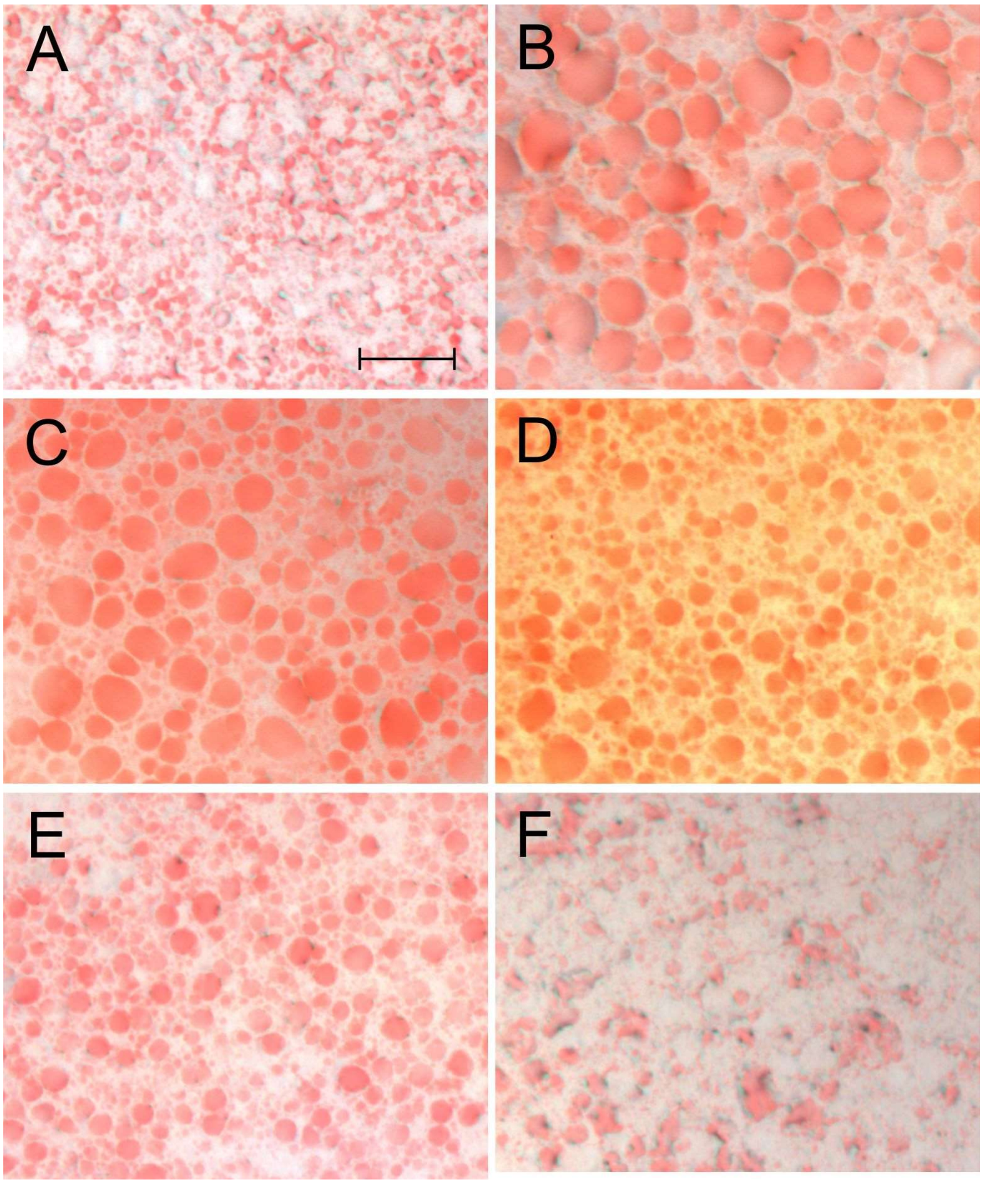

Figure S1. Liver lipid droplets identified by Oil Red O staining. Representative images for lnLA (A), faLA (B), faALA (C), faEPA (D), faDHA (E), and faBASE (F) groups. Scale bar equal to 0.05 mm. *Abbreviations:* faALA, *fa/fa* Zucker rats fed the α-linolenic acid diet; faBASE, baseline *fa/fa* Zucker rats; faDHA, *fa/fa* Zucker rats fed the docosahexaenoic acid diet; faEPA, *fa/fa* Zucker rats fed the eicosahexaenoic acid diet; faLA, *fa/fa* Zucker rats fed the linoleic acid diet; lnLA, lean Zucker rats fed the linoleic acid diet.
